# Essential Role of α5‐GABAA Positive Allosteric Modulation in Cognitive Functions in a Mouse Model of Amyloid Deposition

**DOI:** 10.1002/alz70859_101133

**Published:** 2025-12-25

**Authors:** Jingxin Chen, Carla Elena Mezo‐Gonzalez, Michael D Marcotte, Dishary Sharmin, Prithu Mondal, James M Cook, Etienne Sibille, Thomas D Prevot

**Affiliations:** ^1^ Centre for Addiction and Mental Health, Toronto, ON Canada; ^2^ University of Wisconsin ‐ Milwaukee, Milwaukee, WI USA

## Abstract

**Background:**

Despite extensive drug development efforts, efficacious treatment options for Alzheimer’s Disease (AD) are still lacking. Studies identified the α5‐containing GABAA receptor (α5‐GABAAR) as essential for the regulation of cognitive function and a promising therapeutic target. GL‐II‐73, a positive allosteric modulator at the α5‐GABAAR, showed efficacy at reversing working memory deficits and neuronal shrinkage induced by amyloid load in preclinical models. However, its mechanism of action through the benzodiazepine‐binding pocket of the α5‐GABAARs remains to be demonstrated. This project proposes to show the requirement of positive allosteric modulation of GL‐II‐73 at the α5‐GABAARs in the regulation of cognitive functions in a double transgenic mouse model of AD. We hypothesize that, following chronic treatment, the drug‐sensitive strain will show improved cognitive performance compared to the drug‐insensitive strain, despite both groups having amyloid pathology.

**Methods:**

5xFAD mice were bred with α5‐KI (Knock‐in) mice to generate double transgenic animals. Four‐to‐five‐month‐old double transgenic mice were used, which show amyloid deposition (5xFAD) and insensitivity to drug binding in the benzodiazepine‐binding pocket. Double transgenic animals and their wild‐type littermates (n=12/group, 50% female) underwent three weeks of drug treatment (GL‐II‐73, 30 mg/kg), administered through drinking water. Y‐maze alternation task and Morris Water Maze were conducted to assess working memory and spatial cognition.

**Results:**

Drug‐sensitive mice receiving GL‐II‐73 showed better cognitive performance than the control group in both Morris Water Maze and Y‐Maze alternation task, while such facilitation was not observed in the drug‐insensitive mice.

**Conclusion:**

Our findings demonstrated that the effects of GL‐II‐73 are mediated by allosteric modulation of the benzodiazepine‐binding site of α5‐GABAARs, as expected, specifically at improving working memory and spatial cognition.

